# Case Series of Blowhole Creation with or without Negative Pressure Wound Therapy for Severe Subcutaneous Emphysema

**DOI:** 10.5761/atcs.cr.25-00034

**Published:** 2025-05-27

**Authors:** Toshiko Kamata, Shigetoshi Yoshida, Yuki Hirai, Ryo Karita, Yuki Onozato, Hironobu Wada, Takashi Anayama

**Affiliations:** 1 Department of Thoracic Surgery, School of Medicine, International University of Health and Welfare, Narita, Chiba, Japan; 2 Department of Thoracic Surgery, International University of Health and Welfare, Atami Hospital, Atami, Shizuoka, Japan; 3 Department of Thoracic Surgery, International University of Health and Welfare, Narita Hospital, Narita, Chiba, Japan

**Keywords:** blowhole, negative pressure wound therapy, subcutaneous emphysema

## Abstract

Severe subcutaneous emphysema that is refractory to chest tube drainage can result in significant patient discomfort, airway compromise, and hemodynamic instability. Various interventional approaches, including subcutaneous drain insertion and the blowhole technique, with or without negative pressure wound therapy (NPWT), have been proposed to manage this condition. In this case series, we describe 10 patients who developed severe subcutaneous emphysema following surgery or pneumothorax and were treated using the blowhole technique, with or without NPWT. A Wound Protector/Retractor XXS or LapProtector was used to maintain the patency of the blowhole, facilitating continuous decompression. In cases with more extensive emphysema, the application of NPWT led to rapid respiratory improvement, thereby enabling additional invasive interventions to address the underlying pulmonary air leak. These findings highlight the potential utility of a structured approach incorporating NPWT for the management of severe subcutaneous emphysema, particularly in cases refractory to conventional chest tube drainage.

## Introduction

Subcutaneous emphysema is a common condition that arises following general thoracic surgery, chest tube drainage, or trauma, and it typically resolves spontaneously over time. However, in rare instances, severe subcutaneous emphysema (SSE) accompanied by mediastinal emphysema can develop, leading to extensive skin tightness, palatal closure, dysphagia, impaired eye opening, and airway compromise, which may be life-threatening. The management of SSE that is refractory to conventional chest tube drainage presents a significant clinical challenge. Previous reports have indicated that blowhole creation, either alone or in conjunction with negative pressure wound therapy (NPWT), can facilitate the resolution of SSE and expedite recovery while maintaining ease of application.^1–10)^ This case series describes the use of NPWT in the treatment of SSE.

### Treatment: blowhole creation and NPTW

**Fig. 1:**
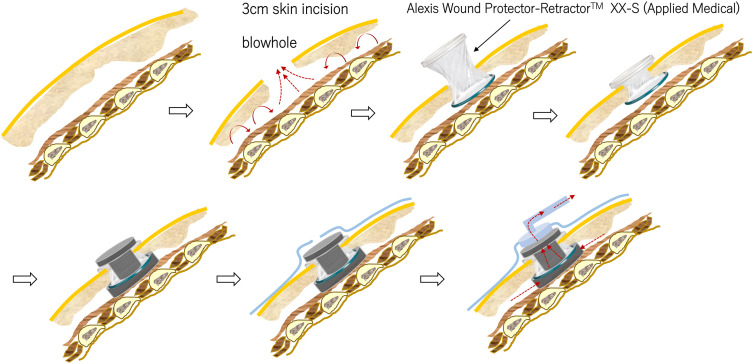
Process of blowhole creation and application of NPWT. A 3-cm skin incision is made caudal to the clavicle, 5 cm below the clavicle, and a Wound Protector/Retractor XXS is inserted to create a blowhole (upper figure). For NPWT, a 1-cm sponge is placed between the Wound Protector/Retractor XXS and the pectoralis major fascia, with an additional sponge covering the top and lumen of the device. The blowhole is then sealed to prevent air leakage, and NPWT is initiated at low pressure (lower figure). Adapted and modified from Fig. 1 in reference 1. NPWT: negative pressure wound therapy

**Fig. 2:**
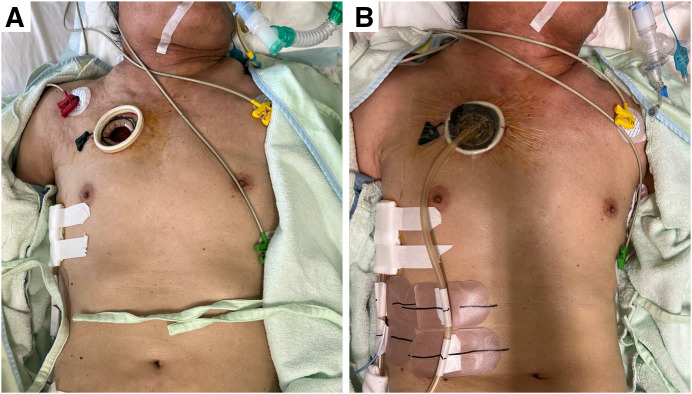
Case of SSE requiring NPWT (**Table 1**, no. 2). A patient with SSE was brought to our hospital under tracheal intubation as a result of inadequate control of SSE following a right chest tube drainage. A skin incision was made on the right anterior chest to create a blowhole (**A**), followed by NPWT application (**B**). NPWT: negative pressure wound therapy; SSE: severe subcutaneous emphysema

Blowhole creation is performed under local anesthesia using the following technique: a 3-cm skin incision is made caudal to the anterior chest, approximately 5 cm inferior to the anterior clavicle. The wound is opened and drained down to the pectoralis major fascia, and a mini wound edge protector—such as the Alexis Wound Protector/Retractor XXS (Applied Medical, Rancho Santa Margarita, CA, USA) or the LapProtector MiniMini (Hakko, Nagano, Japan)—is placed to prevent premature wound closure and minimize contamination (**Fig. 1**).^1)^ This procedure is relatively easy to perform and allows for efficient evacuation of subcutaneous air. Although blowhole creation with a mini wound edge protector device (**Fig. 2A**) enables relatively rapid decompression of subcutaneous air, it may be insufficient in cases of extensive SSE. In such cases, adjunctive NPWT is applied to enhance air evacuation (**Fig. 2B**). The initial suction pressure is set at 50 mmHg and is gradually increased, with a target range of 70–100 mmHg, depending on the case and the patient’s clinical course.[Table table-1]

**Table 1 table-1:** The outcome of blowhole treatment with and without negative pressure wound therapy

Pt. no.	Age/ gender	Disease	Event side	Cause	No. of chest drain	From air leak to blowhole (days)	Blowhole incision	Response	NPWT	Duration of blowhole (days)	Duration of NPWT (days)	Treatment for air leak
1	59/M	Giant bullae	R	Post-OP	1	12	Contralateral	Intermediate	+	13	16	Pleurodesis
2	74/M	Pneumothorax	R	Drainage	1	1	Ipsilateral	Immediate	+	4	4	VATS bullectomy
3	73/M	Malignant pleural mesothelioma	L	Post-OP (P/D)	2	3	Contralateral	Intermediate	+	14	13	—
4	82/M	Pneumothorax	L	Post-OP	1	4	Contralateral	Gradual	+	31	28	Pleurodesis
5	76/M	Lung cancer	R	Post-OP	1	6	Ipsilateral	Immediate		4		Pleurodesis
6	79/M	Pneumothorax	R	Drainage	1	6	Contralateral	Immediate		24		VATS bullectomy
7	73/M	Aspergillus empyema	R	Post-OP	1	24	Contralateral	Intermediate	+	11	11	Muscle flap plombage
8	80/M	Pneumothorax	R	Post-OP	1	7	Contralateral	Intermediate		8		TGF
9	81/M	Pneumothorax	R	Drainage	1	6	Ipsilateral	Immediate		2		VATS bullectomy
10	82/M	Pneumothorax	R	Drainage	2	12	Ipsilateral	Immediate		10		VATS bullectomy

Event side: side of the diseased lung. Blowhole incision: contralateral or ipsilateral to diseased lung. The response is defined as follows: immediate, subcutaneous emphysema was significantly reduced and ocular symptoms improved the following day; gradual, significant improvement took over 1 week; intermediate, improvement was observed between 2 and 6 days. L: left; M: male; NPWT: negative pressure wound therapy; OP: operation; P/D: pleurectomy/decortication; R: right; TGF: thoracographic fibrin glue sealing method; VATS: video-assisted thoracoscopic surgery

## Case Reports

### Patient 1

A 59-year-old man with a history of living donor kidney transplantation and prior right pneumothorax presented with dyspnea. Chest radiography and computed tomography (CT) revealed pneumonia in the right upper lobe, secondary to displacement of the right upper bronchus, caused by a giant bulla in the right lower lobe.

**Fig. 3:**
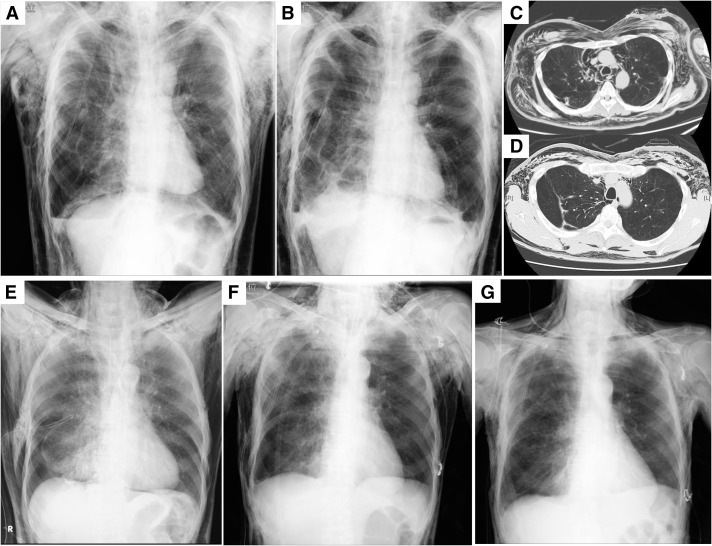
The upper figures illustrate the clinical course of Case 1, while the lower figures depict Case 2. Case 1: Condition on day 7 following resection of giant bullae (**A**), blowhole creation using the Wound Protector/Retractor XXS in the left anterior chest on POD 9 (**B**), 2 days after NPWT initiation (**C**), and 10 days after NPWT initiation (**D**). Case 2: Condition on arrival (**E**), after blowhole creation using the Wound Protector/Retractor XXS on the right anterior chest with NPWT (**F**), and 3 days after NPWT initiation (**G**). NPWT: negative pressure wound therapy; POD: postoperative day

Following giant bullectomy of the right lower lobe, the patient developed a prolonged air leak. Despite several pleurodesis attempts utilizing autologous blood and fibrin glue, SSE and mediastinal emphysema progressively worsened. By postoperative day 7 (POD 7), he exhibited difficulty in opening his eyes, respiratory distress, and tachycardia (**Fig. 3A**). The patient’s inability to tolerate a supine position further complicated additional interventions. On POD 9, a blowhole was created in the left anterior chest using an Alexis Wound Protector/Retractor XXS (Applied Medical) (**Figs. 3B** and **3C**), followed by initiation of NPWT with the RENASYS system (Smith & Nephew, Hull, UK) on POD 12. Clinical improvement was observed, with resolution of skin tightness and respiratory symptoms. On POD 16, a catheter was inserted near the air leak site under CT guidance, allowing for fibrin glue instillation. Subsequently, pleurodesis was reinforced with additional autologous blood and Pichibanil (OK-432, Chugai Pharmaceutical Co., Tokyo, Japan), leading to successful resolution of the persistent air leak. The Alexis Wound Protector/Retractor XXS was removed on POD 21 (**Fig. 3D**), and the chest tube drainage was successfully discontinued on POD 23. Discoloration of the wound edge due to excessive pressure from the wound retractor was evident, so NPWT was maintained for wound healing until POD 27.

### Patient 2

A 74-year-old man with a history of diffuse pulmonary emphysema and cerebral infarction presented to an external hospital with complaints of dyspnea secondary to a moderate right-sided pneumothorax. Following chest tube drainage, the patient experienced rapid expansion of subcutaneous emphysema, leading to progressive airway narrowing and respiratory deterioration, necessitating endotracheal intubation. Consequently, he was transferred to our institution for further management (**Fig. 3E**). Upon arrival, a blowhole incision was created in the right anterior chest using an Alexis Wound Protector/Retractor XXS. NPWT with the V.A.C. system (3M, Maplewood, MN, USA) was initiated immediately (**Figs. 2** and **3F**). The SSE demonstrated progressive resolution, allowing for successful extubation of the endotracheal tube on post-admission day 2. Intensive respiratory rehabilitation was initiated on the same day. On post-admission day 4, the patient underwent video-assisted thoracoscopic surgery (VATS) with bullectomy to definitively treat the pneumothorax. NPWT was discontinued, and the blowhole incision was closed immediately postoperatively (**Fig. 3G**). The chest tube was removed the following day, with no subsequent complications.

### Outcomes

Blowhole incisions were performed in 10 male patients with SSE. Of these, 5 patients received adjunctive NPWT, whereas the remaining 5 experienced resolution of SSE with blowhole incision alone (**Table 1**). In all cases, blowhole incisions were performed within a few days following the onset of ocular occlusion symptoms, with a mean interval of 8.1 days (standard deviation, 6.2 days) from either surgical intervention or chest tube placement to blowhole creation. The blowhole incision was contralateral to the affected lung in 6 patients (66.7%) and ipsilateral in 4 patients. The contralateral side was chosen to avoid infection of the original surgical wound and subsequent thoracic empyema, in accordance with the patient’s medical background. SSE resolved in all cases, and the blowhole remained in place until air leakage had fully ceased. Additional interventions, such as surgery or pleurodesis, were performed in 9 cases to address the air leakage after improvement of the patient’s general status. Among the 5 patients receiving NPWT, the blowhole device was successfully removed, and primary suture closure was achieved within 1–3 days following NPWT discontinuation in all but 1 case. Only 1 patient experienced delayed wound healing attributed to device-related pressure effects, necessitating an additional 3 days of NPWT for wound management prior to extended suture closure.

## Discussion

This report presents a case series evaluating the use of blowhole creation in conjunction with NPWT for the management of SSE. In cases of SSE resulting from persistent air leaks following pneumothorax or pulmonary resection, conventional chest tube drainage may provide only limited therapeutic efficacy, particularly in patients with advanced pulmonary emphysema. This condition, termed recalcitrant subcutaneous emphysema,^4)^ poses significant management challenges. While VATS to repair the damaged pulmonary parenchyma has been shown to substantially reduce hospitalization duration,^11)^ it may not be feasible in the most severe cases due to airway compromise. The insertion of an additional chest tube is frequently employed to enhance thoracic drainage, and the placement of subcutaneous catheters has also been explored; however, failure due to catheter obstruction or dislodgement has been reported.^11)^ Direct skin incision over the affected area alone has limited efficacy, as the wound tends to close prematurely. Prior reports have described the use of packed gauze to prevent premature wound closure, necessitating daily dressing changes and carrying an increased risk of infection.^6)^ Blowhole creation utilizing a mini wound edge protection device designed for endoscopic surgery represents a promising technique for maintaining an open wound while minimizing the risk of premature closure and infection. The Wound Protector/Retractor XXS can be adjusted by rolling it up to match the thickness of the subcutaneous tissue, thereby allowing the creation of a blowhole according to the skin incision. Alternatively, the LapProtector, a more cost-effective option, exerts gentler tension on the tissue; however, its tension cannot be modified through rolling, necessitating additional gauze placement to achieve the desired height in cases of thin subcutaneous tissue. When employing a mini wound edge protector, excessive tension must be avoided to prevent impaired blood flow to the incision site, which may result in pressure necrosis and delayed primary wound healing. Nonetheless, it is important to note that blowhole creation alone is insufficient for managing respiratory and hemodynamic compromise in more severe SSE cases.

The application of NPWT for SSE was 1st reported by Sciortino et al. in 2009,^6)^ using bilateral or unilateral skin incisions over the subclavian region with negative pressures ranging from 50 to 150 mmHg. Subsequent case reports and small-scale case series have been published, with a systematic review in 2022 analyzing 23 cases.^9)^ Although direct application of NPWT sponges to the wound has been associated with risks of bleeding and pain,^9)^ no such complications were observed in the present case series when using a mini wound edge protection device. Our experience in treating elderly patients with SSE demonstrates that blowhole creation combined with NPWT results in a relatively rapid clinical improvement across all cases. Of the 10 patients treated, 5 achieved symptom resolution with blowhole creation alone, while the remaining 5 required adjunctive NPWT, which facilitated respiratory stabilization and permitted subsequent invasive interventions to address persistent air leaks (**Table 1**). NPWT devices, including RENASYS and V.A.C., were selected based on individual patient characteristics and clinical requirements.

Based on our experience in this case series, we propose the following treatment strategy for SSE associated with mediastinal emphysema:

(1) Blowhole creation using a disposable mini wound edge protection device, such as the Wound Protector, should be the 1st-line intervention for symptomatic relief.

(2) NPWT should be introduced if blowhole creation alone proves insufficient in alleviating SSE.

(3) For patients requiring positive pressure ventilation, NPWT should be initiated concurrently with blowhole creation to optimize treatment efficacy.

This report is a retrospective case series from 2 institutions with a limited sample size. Further prospective studies are warranted to validate the efficacy of this approach and optimize treatment strategies for SSE.

## Conclusion

The establishment of a blowhole using a mini wound edge protection device via an anterior thoracic skin incision for SSE represents a straightforward and efficacious intervention. For patients in whom blowhole creation alone does not yield sufficient clinical improvement, NPWT serves as a valuable adjunct for mitigating the symptoms associated with emphysema. This approach is particularly beneficial for patients with SSE who are refractory to management with chest tube drainage alone.

## Acknowledgments

We thank Phoebe Chi, MD, from Edanz (https://jp.edanz.com/ac), for editing a draft of this manuscript.

## Declarations

### Ethics approval and consent to participate

The Ethical Review Committee of Atami Hospital approved this report for publication (Approval No. 24-A-251).

### Consent for publication

Eligible patients were provided the option to opt out.

### Funding

The authors have no funding sources to disclose.

### Author contributions

TK: main author, data collection, and manuscript writing. SY: data collection and final revision of the manuscript. YH, RK, and YO: data collection. HW and TA: data collection and revision of the manuscript. All authors have reviewed and approved the final manuscript.

### Data availability statement

Not applicable.

### Disclosure statement

The authors have no conflicts of interest to declare.
